# The Profiles of Kawasaki Disease in China

**DOI:** 10.2188/jea.11.103

**Published:** 2007-11-30

**Authors:** Tuohong Zhang, Hiroshi Yanagawa, Yosikazu Nakamura

**Affiliations:** 1 School of Public Health, Peking University, P.R. China.; 2 Saitama Prefectural University, Japan.; 3 Department of Public Health, Jichi Medical School, Japan.

**Keywords:** Kawasaki disease, China, Epidemiology, Cardiac sequelae, gamma globulin treatment

## Abstract

Kawasaki disease in China was described for the first time in 1976 in Taiwan, and in 1978 in mainland, respectively. Questionnaire surveys had been conducted in both of the area of China and showed that the Kawasaki disease patients increased year by year. No data on incidence rates were available for these surveys because the problem of representativeness. However, it showed that there were many similar characteristics of Kawasaki disease in China comparing with those in Japan. Although a series of infectious agents were suspected, the etiology of Kawasaki disease remained unclear. High dose of gamma globulin treatment was also adopted commonly in China.

Kawasaki disease (KD) is a disease of unknown etiology affecting most frequently infants and young children under 5 years of age. It was first described in 1967 by Kawasaki in Japan^1^^)^. Fifteen nationwide epidemiological surveys have been conducted in alternate years since 1970^2^^)^. A total of 140,837 patients (81,783 males, 59,054 females; male/female ratio = 1.38) were reported and three outbreaks have occurred in 1979, 1982, and 1986 respectively with the highest incidence of 172-194 per 100,000 children younger than 5 years of age. In the United States, the result of nationwide epidemiological survey showed that hospitalized patients have been accounted to 13,315 cases during the period from1984 through 1990, and the average number per year was 1902 cases. A series of etiological hypothesis emerged but none of them have been identified as etiology of KD. KD has been reported in children of most racial and ethnic groups throughout the world and is now the leading cause of acquired heart disease in children in the United States and in Japan^2^^, ^^3^^)^. There were other different features between Japanese and Chinese observed in spite of general similarities in some aspects. We will overview the profiles of KD in Mainland China, Hong Kong, and Taiwan. This review highlights the insights that have been gained and the challenges that remain in the study of this disease in China.

## EMERGENCE KAWASAKI DISEASE IN CHINA AND ITS CLINICAL FEATURES

Kawasaki disease has been reported for the first time in Taiwan in 1976^4^^)^ and in Shanghai of Mainland China in 1978^5^^)^. Hu YJ reported 11 cases hospitalized in large cities in 1979 with typical clinical manifestation of KD^6^^)^. Fifty-eight cases (36 males and 22 females, 71% were under 3 years of age) during the period from March 1975 through February 1985 admitted in Beijing Children’s Hospital had been observed on the aspects of cardiovascular lesion and treatment^7^^)^. They found 45% of cases with cardiovascular lesion by using 2D-echocardiography, X-ray and electrocardiography.

Forty-six typical cases were reported in Taiwan from 1976 through 1983^8^^)^. A total of 187 atypical cases from 1983 through 1992 were also discussed for unusual manifestation such as transient thrombocytopenia and isolated azotemia^9^^)^. They also found that there was no difference in clinical manifestations among the patients younger than 6 months compared with the patients older than 6 months of age^10^^)^. However, the diagnosis was generally delayed for those of atypical clinical features.

Physical involvements other than coronary artery lesions (CAL) were reported with colon involvement, for instance, the ischemic colitis in Hong Kong^11^^)^ as well as in Taiwan^12^^)^ with disseminated intravascular coagulation. The former was successfully treated with aspirin and the latter with high dose gamma globulin. There were but rare cases emerged in Taiwan as Reye syndrome^13^^)^, small bowel pseudo-obstruction^14^^)^, and gallbladder hydrops mimicking acute abdomen^15^^)^.

## EPIDEMIOLOGY

Epidemiological surveys have been carried out both in Taiwan and in the mainland early in 1980’s. From 1983 through 1992, three nationwide surveys have been conducted with 100 hospitals in selected large municipalities of the mainland participated and total of 3,991 KD patients have been reported during the 10 years^16^^-^^18^^)^. Pediatricians were responsible for these investigations without the collaboration of epidemiologists. They surveyed in the year during 1983-1986, 1987-1991 and in 1992.

The diagnostic guidelines (4^th^ edition) published by Japan Kawasaki Disease Research Committee were adopted in the surveys and the results were obtained through mail survey forms. Patients increased year by year and the number of patients in 1992 was 5.4 folds of the number in 1983 (Table 1). Large proportion of patients had been found in the Children’s hospitals in main developed cities than in the other developing inland cities. Very few patients had been found in the large hospitals in remote provinces.

**Table 1.** The proportion of reported KD patients by year.

Year	Number	%
1983	139	3.5
1984	179	4.5
1985	244	6.1
1986	277	6.9
1987	360	9.0
1988	410	10.3
1989	459	11.5
1990	566	14.2
1991	612	15.3
1992	745	18.7
Total	3991	100.0

Of the total cases of 3,991 patients, 2,452 were males and 1,539 were females. The male/female ratio was 1.6:1 and varied from 1.2 to 1.8 in different years. In Japan, the ratio is 1.4:1 and the proportion of male patients was smaller than usual in epidemic years. Patients younger than 2 years of age accounted for 43.1%, those older than 2 years of age and younger than 4 years accounted to be 36.7%. This proportion was smaller than in Japan, where the proportion of patients younger than 5 years of age is more than 80%. Two fatal patients were reported during tire period from 1983 through 1986 (0.24%) and 8 during the period from 1987 through 1991 (0.33%). In 1992, among 745 patients, 98 received combination treatment of gamma globulin and aspirin (13.2%), 529 received combination treatment of aspirin and panthetin (71.0%) and 71 received combination of aspirin and prednisone^16^^)^.

The diagnosis of coronary involvements during the period of 1983-1986 had been simply made according to clinical features and judged by the pediatricians only. Therefore, the number of patients who had been judged as with coronary involvements was fewer than during the period of 1987-1991 (Table 2). The inner size of the coronary artery had been recorded when it was found to have dilatation since 1987, and it was recognized as cutoff point for the seriousness of cardiac sequelae (Table 3).

**Table 2.** The number of patients who have coronary involvement.

	1983-1986	1987-1991	1992
Diagnosis base	Clinical features	Echocardiography	Echocardiography
Total No. of cases	839	2417	745
Case with CAL	34	291	177
%	4.1	12.0	23.0

CAL : coronary artery lesion

**Table 3.** Severity of coronary involvement.

Inner size of coronary artery (mm)	Mild 3-4	Medium 5-8	Serious overthan8
Number of case	101	58	12
%	59.1	33.9	7.0

Eleven of 100 hospitals had followed up 188 cases with cardiac involvements for 1 to 5 years for observing the progress of the cardiac sequelae. Cardiac involvements disappeared in 146 (77.6%) cases, abbreviated in 20 (10.6%) cases, remained unchanged the inner size of the artery in 13 (6.9%). Only 4.8% of the cases followed had worse results: 6 became worse wife the coronary dilatation larger, and 3 died.

Epidemiological research was carried out in Taiwan as well^4^^)^. In Taiwan, 1,042 cases have been reported from 1976 through 1986 in all the 18 hospitals. The number of cases increased year by year, with 3 cases in 1976, 12 in 1977 and 16 in 1978. By the end of 1981, the number of patients dramatically increased to 36, and 92 in 1983, 163 in 1984, 438 in 1986, respectively. The occurrence was characterized with stability among seasons though with slightly increases from April to June. The age of new cases predominated below 2 years, accounted to 65%. Proportion of patients with coronary artery aneurysms was 31.5% (286/907), and the case-fatality rate was 0.4% (4/1047).

A five-year period retrospective survey was conducted in Hong Kong recently^19^^)^. During the period from 1 July 1994 through 30 June 1999, 572 cases of KD were reported. The average yearly incidence rate was 31.0 per 100,000 children younger than five years of age. There are 354 males and 218 females among the patients, the male: female ratio was 1.6:1. The median age of them is 1.6 years, 31.8% was infant. The occurrence of the disease spread evenly over the five years with peak at late spring and summer, the wet season. Coronary artery aneurysms or dilatation were present at the 4^th^ week in 58/572 (10.1%) of children.

Very few epidemiological surveys have been carried out in China. It was because some pediatricians lacked knowledge about diagnosis of Kawasaki disease, especially among the pediatrician in remote areas.

Compared with the conditions in Japan, the occurrence of KD was literally few in China. However, it was clear that the patients increased year by year. In recent years, Kawasaki disease became one of the most important cause of postnatal (acquired) heart disease by taking the place of rheumatic fever as well.

There are evidences that early treatment by gamma globulin will decrease the risk of coronary lesions. It is necessary to make an early diagnosis for patients who suffered from Kawasaki disease. Hence pediatricians are needed to know much about it through deep education in China, especially in remote areas.

Previous results of epidemiological surveys have cleared out some features on Kawasaki disease. However, these works failed to make the data of cross-cultural comparability and to calculate the incidence rate among the population because of the problem in representativeness.

## ETIOLOGY AND PATHOLOGY

Many etiologic agents have been suspected, including streptococcus, rickettsia, virus, other unknown microorganisms, mite-transmitted agents, toxic substances of pharmacological or of chemical nature, and allergic reactions, but none of these has yet been confirmed.

Shen^20^^)^ excluded possibility of streptococcal infection by the bacteria through a case-control study in Taiwan that cultured throat swab of Kawasaki Disease patients. The control group was patients with respiratory diseases. Tang^21^^)^ showed no relationship between mite antigen and its specific responsive IgE and IgD among patients in Taiwan. Chang^22^^)^ showed no association of HLA antigen with KD in Hong Kong.

Pathological observations on skin lesions in 2 cases of KD in the Mainland^23^^)^ showed spiral body existed as susceptible etiology of KD. Virus-like particles with reverse transcriptase (RT) activities were found associated with KD^24^^)^.

The etio-pathogenesis remains unknown. It is known that immune responses are predominantly activated in KD patients during the acute phase. Several laboratory findings are summarized as shown in Table 4.

**Table 4.** Selected laboratory findings among KD patients in China.

	Author	Year	Location	Research design	Clinical findings
1	Chiang AN	1997	Taiwan	Randomized Clinical Trail	HDL(+) Trigliceride(+)
2	Ding X	1993	Mainland	Case-control (62/100)	lgG,BCGF,IL-6, TNF(+)
3	Lin CY	1991	Taiwan	Case report	IL-2(+)
4	Li C	1990	Mainland	Case-control (23/21)	lgG-CIC(+)
5	Lin CY	1987	Taiwan	Case report (20)	IgG-CIC, IgG(+)

## DIAGNOSIS

Two-dimensional echocardiography (2DE) has been widely applied in Mainland^25^^, ^^26^^)^ and in Taiwan^27^^-^^29^^)^ as well as in Japan. 2DE remains as a predominant method for diagnosis of KD in the mainland although there are other alternatives in Taiwan, such as 99 Tcm-HMPAO-labelled WBC scanning^30^^)^ and EMBPV (Equilibrium Multigated Blood Pooling Ventriculography)^31^^)^.

Marked redness over BCG vaccination site had also been observed in Chinese patients^32^^-^^33^^)^.

Cardiac sequelae were especially discussed because it was the most harmful prognosis related with KD patients. Researchers in Taiwan found IgG-CIC, PGE2^34^^)^, IL-6, IL-8^35^^)^, tumor necrotic factor (TNF), Interleukin-2 (IL-2), interferon-gamma (IFN-r)^36^^)^ could be used as predictors of coronary arterial lesions. The combination of 2 parameters WBC count and C-reaction protein^37^^)^ was suggested to be applied as early identifying indicator of KD patients.

## TREATMENT

Aspirin and gamma globulin were the two predominant drugs for treatment of KD patients, especially when they developed coronary arterial lesions.

Liang^7^^)^ reported 58 patients were successfully treated with aspirin, steroid hormone and the combination of anti-biotic with traditional herb drug in mainland. In Taiwan, patients who received aspirin without administration of gamma globulin tended to be susceptible with coronary arterial lesions^38^^)^.

Hwang^39^^)^ reported 106 cases that were treated in 4 regimens. Regimen I was the combination of aspirin with 130-200mg/kg body weight of intravenous gamma globulin, regimen II was the combination of aspirin with 201-400mg/kg of intravenous gamma globulin, regimen III was aspirin alone and regimen IV was no treatment. The result showed that even with delay in the time of start of prophylactic gamma globulin therapy, it still reduced the formation of giant coronary aneurysm. In Japan, 400mg/kg/day of gammaglobulin for 5 days is the commonest, but nowadays 2000mg/kg for one day becomes prevalent as well as in the United States.

Wu^40^^)^ and Hwang^41^^)^ also reported 293 cases that were treated in 3 different regimens. Regimen I was aspirin alone, regimen II was the combination of aspirin with moderate intravenous gamma globulin (400mg/kg for 5 days), regimen III was the combination of aspirin with high single dose of gamma globulin (2000mg/kg). The result showed that gamma globulin initiated within 10 days of the onset of fever, in conjunction with aspirin decreased the prevalence of coronary artery dilatation and aneurysms significantly in comparison with treatment by aspirin alone. However, there was no difference in the prevalence of coronary aneurysm between the group of single high dose and multiple doses of gamma globulin, though the single high dose of gamma globulin can improve the clinical symptoms quickly and shorten the duration of hospitalization.

Clinical trial was carried out on the patients without coronary abnormalities within 10 days of the onset^42^^)^. Out of 291 patients, 128 cases were treated with gamma globulin (400mg/kg for 4 consecutive days) with the combination of aspirin, and 163 were treated with aspirin alone. It showed that gamma globulin was effective in reducing the prevalence of coronary artery abnormalities. Yang^43^^)^ also identified successful treatment by reducing the prevalence of coronary arterial lesion with high-dose intravenous gamma globulin treatment. He carried out a prospective 2DE study on coronary arterial lesions in 109 KD patients during the period from August 1983 to March 1990.

However, some studies related to gamma globulin treatment showed no evidence on significant differences in the parameters of left ventricular function^44^^)^, the improvement of already existed carditis and dilated coronary arteries before and after treatment^45^^)^.

## FUTURE CONSIDERATION

We strongly suggest that fruitful avenues for future researches in China include the following: 1) epidemiological investigations of the national or provincial incidence rate of the disease; 2) intensive and comprehensive clinical retrospective study; 3) follow-up of patients into their adolescent, and adulthood if possible; 4) expanded studies of the treatment; 5) increase national and international collaboration as well as the training of qualified doctors.

## References

[1] Kawasaki T. Acute febrile mucocutaneous syndrome with lymphoid involvement with specific dequamation of the fingers and toes in Children (in Japanese). Jpn J Allergy 1967; 16: 178-222. 6062087

[2] Yanagawa H, Nakamura Y, Ojima T, Yashiro M, Tanihara S, Oki I. Changes in epidemic patterns of Kawasaki disease in Japan. Pediatr Infect Dis J 1999; 18:64-66. 9951983 10.1097/00006454-199901000-00015

[3] Taubert KA, Rowley AH, Shulman ST. A nationwide survey of Kawasaki disease and acute rheumatic fever. J Pediatr 1991; 119: 279-282. 1861216 10.1016/s0022-3476(05)80742-5

[4] Lue HJ. An epidemilogical survey on Kawasaki disease in Taiwan. Acta Paediatr Sin 1987; 28:72.

[5] Yu SC. Mucocutaneous lymph node syndrome (10 cases report). Shanghai Med 1978; (5):24.

[6] Hu YJ. Mucocutaneous lymph node syndrome (11 cases report). Chinese Pediatr J 1979; 17:107-110.

[7] Liang YC, Jin H, Han TZ, Zhang XC, Mo HG. Coronary artery changes in Kawasaki disease. Chinese Circul J 1987; (8): 459-461.

[8] Yang HY, Lin GJ, Lee CY, Lue HC. Clinical observation of mucocutaneous lymph node syndrome. Acta Paediatr Sin 1985; 26: 213-222.

[9] Huang YC, Lin TY, Su WJ. Unusual manifestations in children with Kawasaki disease. J Foros Med Assoc 1997; 96: 451-456. 9216170

[10] Hsiao JY, Chen MR, Huang FY, Kao HA, Sung TC. Clinical analysis of Kawasaki disease in infants below 6 months of age. Acta Paediatr Sin 1988; 29: 318-323. 3272533

[11] Fan ST, Lau WY, Wong KK. Ischemic colitis in Kawasaki disease. J Pediatr Surg 1986; 21: 964-965. 3794953 10.1016/s0022-3468(86)80107-5

[12] Huang LW, Sun W, Huang FY. Kawasaki disease presenting with disseminated intravascular coagulation: report of one case. Acta Paediatr Sin 1994; 35: 341-344. 8085458

[13] Lee JH, Hung HY, Huang FY. Kawasaki disease with Reye syndrome: report of one case. Acta Paediatr Sin 1992; 33: 67-71. 1626454

[14] Huang YC, Ho MY, Huang FY, Hsu JC. Kawasaki disease complicated with hemorrhagic enteritis mimicking intestinal obstruction: report of one case. Acta Paediatr Sin 1990; 31: 53-57. 2278230

[15] Hou JW, Chang MH, Wu MH, Lee CY. Kawasaki disease complicated by gallbladder hydrops mimicking acute abdomen: a report of three cases. Acta Paediatr Sin 1989; 30: 52-60. 2700275

[16] Liang YC, Wang NK, Zhao XJ. Incidence survey on Kawasaki disease among hospitalized patients in 78 hospitals during from 1987 to 1991. Clin Pediatr 1994; 12: 319-320.

[17] Liang YC, Wang NK, Chai XM. Overview of Kawasaki disease in China. J Chinese Prac Pediatr 1995; 10: 302-303.

[18] Zhang SR. Etiology and epidemiologic research on children’s Kawasaki disease. Med Res Communi 1996; 25: 24-25.

[19] Ng YM, Sung RYT, So LY, Ho MHK, Cheng YW, Chan KC, et al. Acquired Heart Disease. The Abstracts of 10^th^ Asian Congress of Pediatrics, Taipei, Taiwan, March 26-30, 2000.

[20] Shen CT, Wu HY, Wang NK, Huang CS. Reevaluation of streptococcal infection in the pathogenesis of Kawasaki disease. Acta Paediatr Sin 1990; 31: 144-150. 2275373

[21] Tang RB, Hwang B, Tsai LC, Lin FM, Chang HN. The significance of mite antigens in Kawasaki disease. Chinese J Microbiol Immunol 1987; 20: 29-36. 3595263

[22] Chang CC, Hawkings BR, Kao HK, Chow CB, Lau YL. Human leucocyte antigens in southern Chinese with Kawasaki disease. Eur J Pediatr 1992; 151: 866. 1468468 10.1007/BF01957943

[23] He ZF, Li S, Zhou JY, Hong T, Qin ZT. Pathological observations on skin lesions in 2 cases of Kawasaki syndrome. J Chinese Pathol 1986; 15: 228-229. 2953467

[24] Lin CY, Chen IC, Cheng TI, Liu WT, Hwang B, Chiang BN. Virus-like particles with reverse transcriptase activity associated with Kawasaki disease. J Med Virol 1992; 38: 175-182. 1283752 10.1002/jmv.1890380305

[25] Zhu Q, Tang SC, Yang F. Multi-aspects observation of 2D-echocardiography on coronary artery lesions of Kawasaki disease. J Chinese Cardiovasc Dis 1988; 16: 289-90.

[26] Liang Y, Han TZ, Zhu JC, Li T, Wu MC, Du SZ. Clinical analysis on 58 cases with MCLS. Beijing Med 1987; 9: 132-134.

[27] Hwang B, Wang DH, Meng CCL. Electrocardiographic and echocardiographic studies in children with Kawasaki disease. Acta Paediatr Sin 1984; 25: 369-376.

[28] Wu MH, Lue HC, Cheng SJ, Sung TC. Echocardiographic visualization of coronary arterial lesion in Kawasaki disease. Acta Paediatr Sin 1986; 27: 1-9.

[29] Kao CH, Chen YC, Hsieh KS, Wang SJ, Yen SH. Equilibrium multigated blood pool ventriculography to evaluate the effects of high-dose gamma-globulin treatment for left ventricular functions in Kawasaki disease. Clin Nucl Med 1994; 19: 723-726. 7955755 10.1097/00003072-199408000-00017

[30] Kao CH, Hsieh KS, Wang YL, Chen CW, Liao SQ, Wang SJ, Yen SH. Comparison of 99TCm-HMPAO-labeled white blood cells and 67Ga citrate scans to detect myocarditis in the acute phase of Kawasaki disease. Nul Med Communi 1991; 12: 951-958. 10.1097/00006231-199111000-00004 1754155

[31] Kao CH, Hsieh KS, Wang SJ, Yen SH. Evaluation of left ventricular function in Kawasaki disese using equilibrium multigated blood pooling ventriculography. Clin Nucl Med 1995; 20: 263-266. 7750223 10.1097/00003072-199503000-00016

[32] Chen CY, Chen LC. Marked redness over BCG vaccination site in a case of mucocutaneous lymph node syndrome. Abstracts of 105th Conference of Pediatrician Association. 1986.

[33] Hsu YH, Wang YH, Hsu WY, Lee YP. Kawasaki disease characterized by erythema and induration at the Bacillus Calmette-Guerin and purified protein derivative inoculation sites. Pediatr Infect Dis J 1987; 6: 576-578. 3615073

[34] Lin CY, Hwang B, Chiang BN. High risk factors in the development of coronary aneurysms in patients with mucocutaneous lympy node syndrome(Kawasaki disease). Acta Cardiol Sin 1986; 2: 253-262.

[35] Lin CY, Lin CC, Hwang B, Chiang BN. Cytokines predict coronary aneurysm formation in Kawasaki disease patients. Eur J Pediatr 1993; 152: 309-312. 8482278 10.1007/BF01956740

[36] Lin CY, Lin CC, Hwang B, Chiang BN. The changes of interleukin-2, tumour necrotic factor and gamma-interferon production among patients with Kawasaki disease. Eur J Pediatr 1991; 150: 179-182. 1904353 10.1007/BF01963561

[37] Lu CP, Lee WJ, Ho MM, Hwang KC. Risk factors of coronary arterial aneurysm in Kawasaki disease. Acta Paediatr Sin 1993; 34: 173-180. 8368064

[38] Tzen KT, Wu MH, Wang JK, Lue HC, Lee CY, Chien SC. Prognosis of coronary arterial lesions in Kawasaki disease treted without intravenous immunoglobulin. Acta Paediatr Sin 1997; 38: 32-37. 9066187

[39] Hwang B, Lin CY, Hsieh KS, Tsuei DH, Meng CCL. High dose intravenous gamma globulin therapy in Kawasaki disease. Acta Paediatr Sin 1989; 30: 15-22. 2484055

[40] Wu JR, Hwang KP, Tu JG, Dai ZK, Huang TY. Study on coronary artery lesions in patients with Kawasaki disease: recent 9 years’ experience. Kaohsiung J Med Sci 1993; 9: 27-38. 7682261

[41] Hwang KP, Wu JR, Huang LY, Liou CC, Huang TY. Clinical manifestations and effects of IVGG in patients with Kawasaki disease. Kaohsiung J Med Sci 1996; 12: 159-166. 8709183

[42] Hsu CH, Chen MR, Hwang FY, Kao HA, Hung HY, Hsu CH. Efficacy of plasmin-treated intravenous gamma-globulin for therapy of Kawasaki syndrome. Pediatr Infect Dis J 1993; 12: 509-512. 8345983 10.1097/00006454-199306000-00010

[43] Yang CC, Lue HC, Wang JK, Wu MH, Wu YN. A detection and follow-up study of coronary arterial lesions in Kawasaki disease by two-dimensional echocardiography. Acta Cardiol Sin 1990; 6: 262-275.

[44] Kao CH, Hsieh KS, Chen YC, Wang YL, Wang SJ. Relationships between coronary artery dilatation and severity of carditis detected by two-dimensional echocardiography and [99mTc] HMPAO-labeled white blood cell hear scan in children with Kawasaki disease. Pediatr Radiol 1994; 24: 41-44. 8008494 10.1007/BF02017659

[45] Kao CH, Hsieh KS, Chen YC, Wang YL, Wang SJ, Yen SH. Labeled WBC cardiac imaging and two-dimensional echocardiography to evaluate high-dose gamma globulin treatment in Kawasaki disease. Clin Nucl Med 1995; 20: 813-816. 8521660 10.1097/00003072-199509000-00012

